# Supplementary material for: Elites, communities and the limited benefits of mentorship in electronic music
Source: Sci Rep. 2020 Feb 21;10:3136. doi: 10.1038/s41598-020-60055-w (PMC7035280; doi:10.1038/s41598-020-60055-w)
Supplement: Supplementary file 1 — Supplementary Information. [file 41598_2020_60055_MOESM1_ESM.pdf]

Supplementary Information for

Elites, communities and the limited benefits of mentorship in electronic music

Milán Janosov[1], Federico Musciotto[1], Federico Battiston[1], and Gerardo Iñiguez[1, 2, 3, *]

[1]Department of Network and Data Science, Central European University, Budapest, 1051, Hungary

[2]Department of Computer Science, Aalto University School of Science, Aalto, 00076, Finland

[3]IIMAS, Universidad Nacional Autonóma de México, Ciudad de México, 01000, Mexico

[*]iniguezg@ceu.edu

S1 Dynamics of the top 100 ranking list

S1.1 Is the top 100 the real top?

In addition to the study of rank diversity in Section 3.1, we define and compare the following two measures of the ranking list in order to show the difference between the top and the bottom of the ranking (results are also shown on Figure S1):

Jaccard similarity across years. We compare set of DJs being present at rank r at a given year t ($P_r(t)$) to the DJs in r at time $t+1$ ($P_r(t+1)$), average this over time, and study it as a function of r (Figure S1a):

$$\overline{J_r} = \left\langle \frac{P_r(t) \cap P_r(t+1)}{P_r(t) \cup P_r(t+1)} \right\rangle_t . \qquad (1)$$

By analyzing the behavior of this measure as a function of r (Figure S1a,c), we see a regime change from a monotounsly decreasing trend to a flat line around $r^* \approx 20-25$, which overlaps

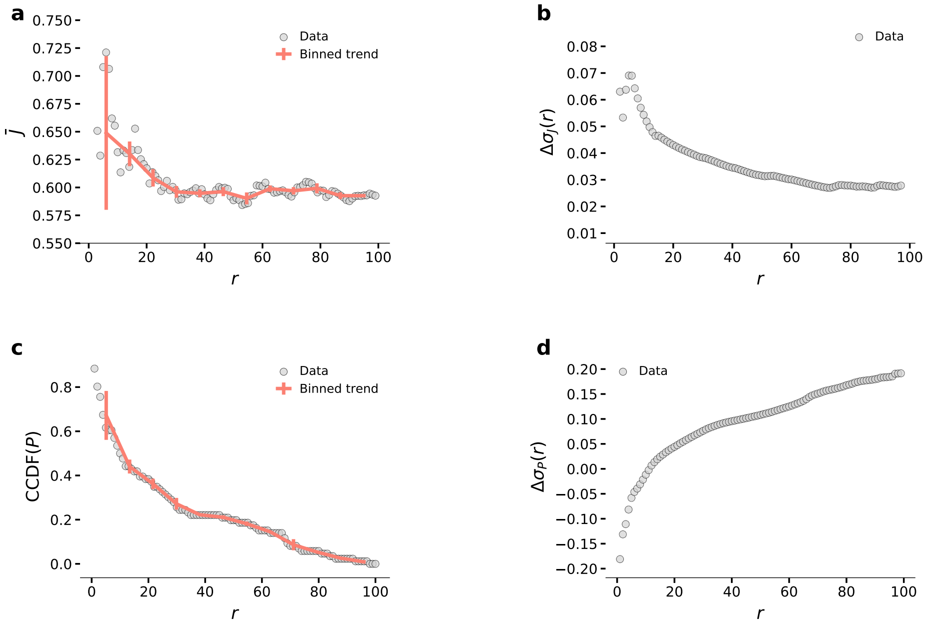

Figure S1. Homogeneity of the ranking. The (**a-b**) subfigures show the Jaccard similarity $\bar{J}$ and its splitting variance based on Eq. 1 in the main text, while (**c-d**) visualize the CCDF of the remaining probability P as a function of the splitting rank r and its splitting variance, further pointing out the differences between the top and the bottom of the ranking.

with our finding presented in Section 3.1. The same trends are also visible on the variance differences if we split the ranking at r according to Eq. 1 in the main text (Figure S1b,d).

Remaining probability. The probability P of an individual taking the same rank position at time t and time $t+1$. For a certain rank r we compute the number of times the same individual occupies that rank at time t and $t+1$, and average it over time. Figure S1c shows the complementary cumulative distribution function (CCDF) of this probability as a function of r and shows that there is a clear change in trend around the 20th position, further supporting that neither top 10 nor top 100 are special; however, somewhere in-between there is a clear separation between a stable, popular elite, and the rest of musicians. We also measure the splitting variance difference (Eq. 1 in the main text), which shows a change in trend around $r^* \approx 20$ as well (Figure S1d).

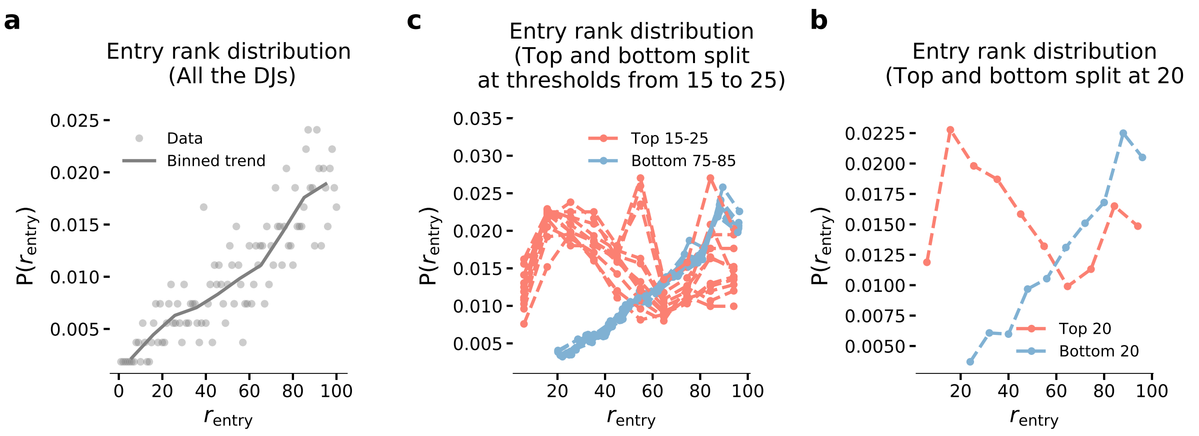

Figure S2. Entry rank distribution. (**a**) The entry rank distribution of the total population. (**b**) The entry rank distribution of the top and bottom DJs, split based on their best rank with different splitting thresholds (from 15 to 25). (**c**) The entry rank distribution of the real top DJs and the rest of the ranking.

S1.2 How does the real top differ from the rest?

Here we compare the distribution (Figure S2a) of the entry rank of the DJs over the entire population, and at various splitting thresholds, including splitting between the identified real-top boundary (Figure S2b-c). As for the distribution of entry ranks, we see that there are increasingly more people entering the ranking at lower ranks. However, if we split individuals at the threshold of the real top, we see noisy but quite different trends. Having said that, the trends seem to be inverted: while 'bottom' individuals are increasingly more likely to enter at lower ranks, high achievers are more likely to enter at higher places immediately.

S1.3 Relationship between the best ranks and the songs' raw popularity

The top 100 ranking is based on the number of votes coming from the fans of DJs. The raw vote counts and ranks below 100 are not available. We compare the results of last year (2018) in the following way. We consider those DJs who were present in the top 100 in 2018 (announced on 20 October 2018). Then we pick all the songs of these DJs that were released before the ranking

	December 20	February 17	March 22	Apr 19	May 26
Total play count of songs in 2018	14,669,041	17,870,288	27,181,518	17,831,911	18,007,282
Corr(Rank last year vs. play count of songs released in 2018)	0.394 $^{(0.0091)}$	0.393 $^{(0.0008)}$	0.371 $^{(0.0007)}$	0.346 $^{(0.0017)}$	0.35 $^{(0.0015)}$
Corr(Best rank ever vs. total play count over the career)	0.262 $^{(3.3\cdot10^{-6})}$	0.321 $^{(3.2\cdot10^{-6})}$	0.332 $^{(8.7\cdot10^{-8})}$	0.318 $^{(4.8\cdot10^{-7})}$	0.319 $^{(4.2\cdot10^{-7})}$

Table S1. Play count and ranking correlations. Spearman rank correlation values (and related p-values) between number of songs (released in 2018–2017) and the ranking of 2018; play counts of songs released during several 2-3 day-long crawling periods (marked by their start dates); and correlation between the best rank of DJs and the overall play count of their songs. The decreasing trend of total play counts shows how songs released in 2018 are losing popularity in 2019.

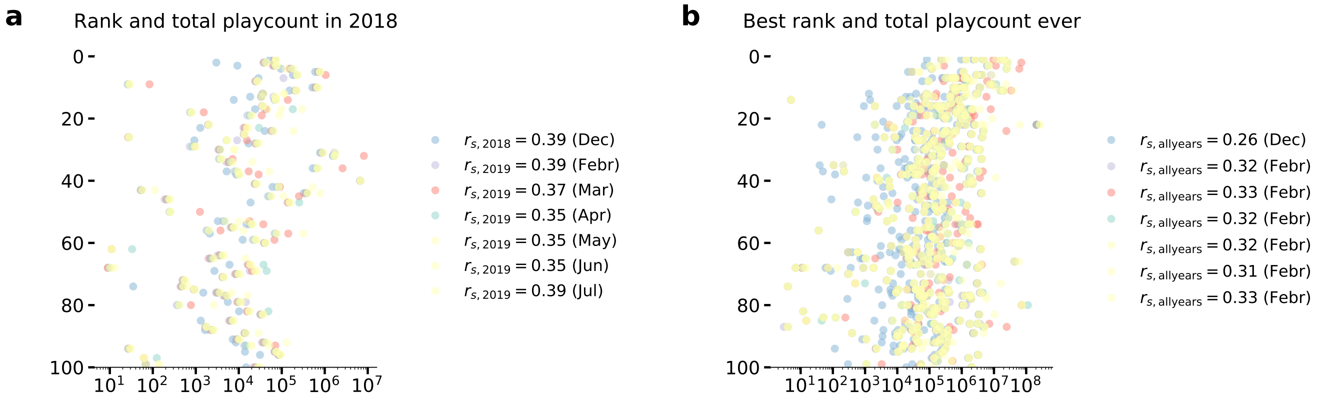

Figure S3. Absolute values of the computed Spearman-rank correlations between songs' popularity and top 100 ranking. (**a**) Correlation plot between the ranks of DJs (in 2018) and the total play count of their songs released during the year before this ranking. (**b**) Correlation between the total play count of the songs ever released by these DJs, and their best rank on the top 100 ranking ever.

of 2018 came out, but after the ranking of 2017 came out, covering a period of one year. Then we compute the overall popularity (total play count) of these songs at different points in time (as detailed in Figure S3) and compute their correlations with the overall ranking. We also correlate the best rank ever achieved by the top DJs to the total play count of the songs they released (Figure S3b), where we find an even lower correlation. The correlation values are summarized

	Degree	Betweenness	PageRank	Clustering
Best rank	0.263	0.234	0.261	0.109
Average rank	0.207	0.172	0.202	0.09

Table S2. Correlation between the different centrality measures and the best and the average ranks of the DJs.

in Figure S3) and Table S1.

S2 Co-release network in the world of electronic music

S2.1 Network visualization

To obtain the network visualization on Figure 2a we go through the following steps. First, we construct the (quite dense) original sub-network of top 100 DJs, with 15,403 edges distributed among 486 nodes in the giant component. Then we apply network filtering algorithms before further analysis. We use the recently introduced noise-corrected filter method,[1] with which we filter out $\sim 88\%$ of edges while keeping $\sim 86\%$ of nodes. To extract communities we use an established heuristic method.[2]

S2.2 Network centralities and success

Here we show the correlations between the best and average ranks that the top 100 DJs have achieved and their measured network centralities (captured by degree, betweenness, and PageRank centrality), as well as the clustering coefficient. The results, highlighting a surprisingly low correlation are shown in Figures S4-S5 and summarized in Table S2.

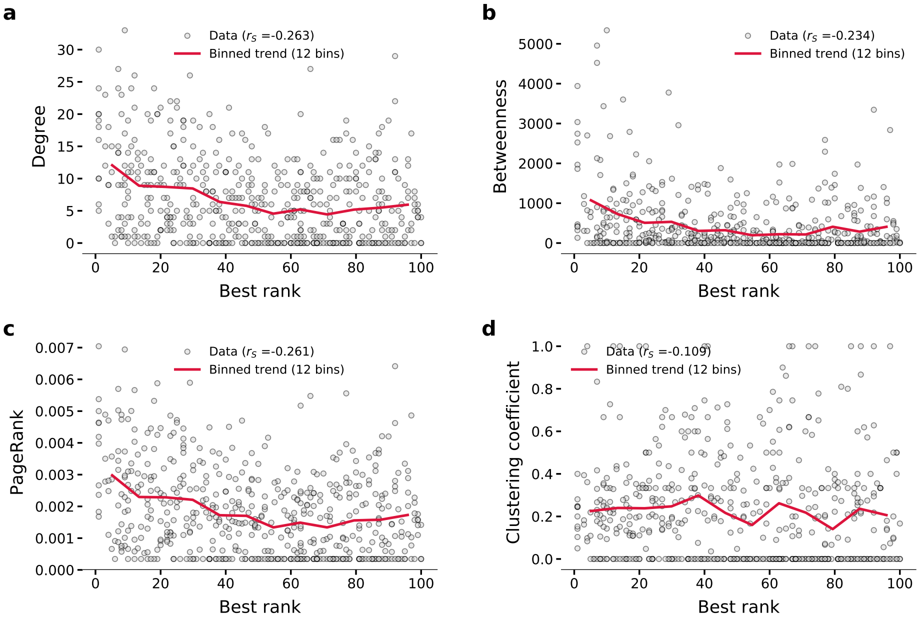

Figure S4. Correlations between the best rank and network centralities. The red line visualizes the binned trends of the centralities (node degree, betweenness centrality, PageRank centrality, and clustering), while individual points represent individual DJs.

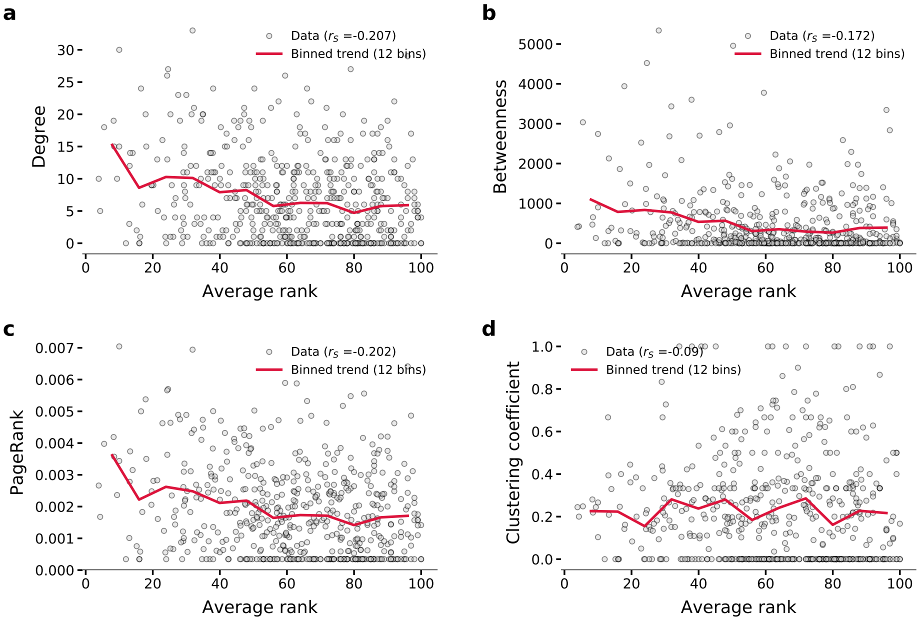

Figure S5. Correlations between the average rank and the network centralities. The red line visualizes binned trends, while individual points represent individual DJs.

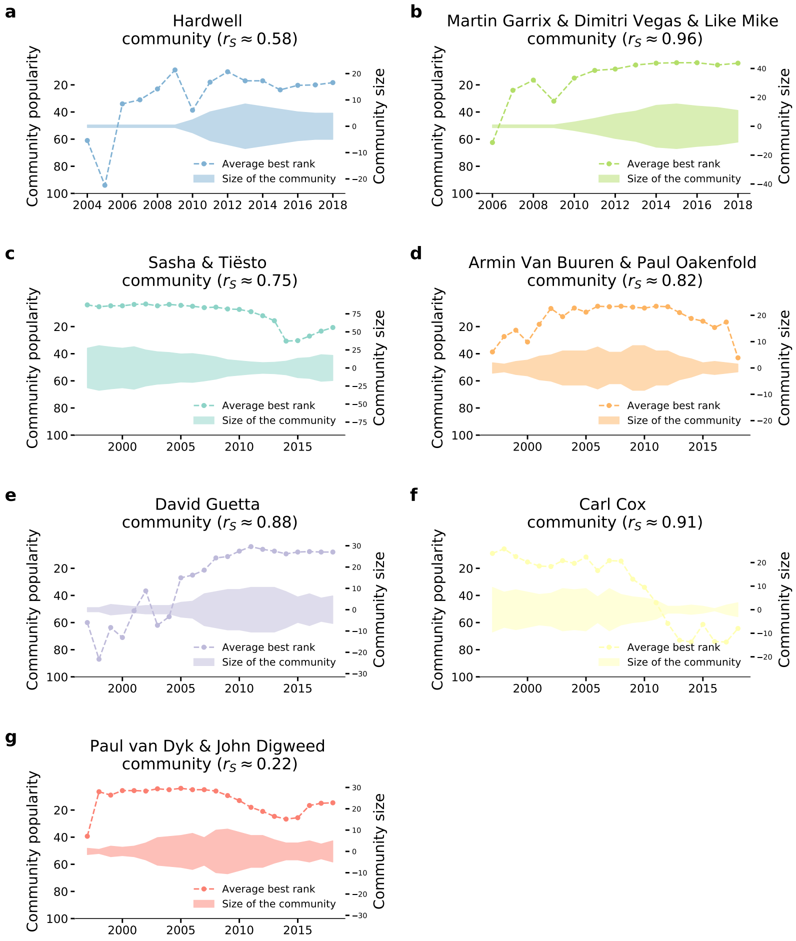

Figure S6. Temporal dynamics of communities in electronic music. Average popularity of the three highest-ranked DJs of each community (colored dashed lines), and size of the community (shaded area over time). Titles include the Spearman rank correlation r_s between the two quantities over time.

S2.3 Communities over time

On Figure S6 we show how the size of DJ communities changes over time, and how it correlates with the popularity of the communities over time. We quantify popularity by taking the average rank of the three highest ranked DJs of each of the seven largest communities of the giant component of the top 100 DJ's network. The figures show high correlation, implying that the more popular a community is, the more members it has in the top 100.

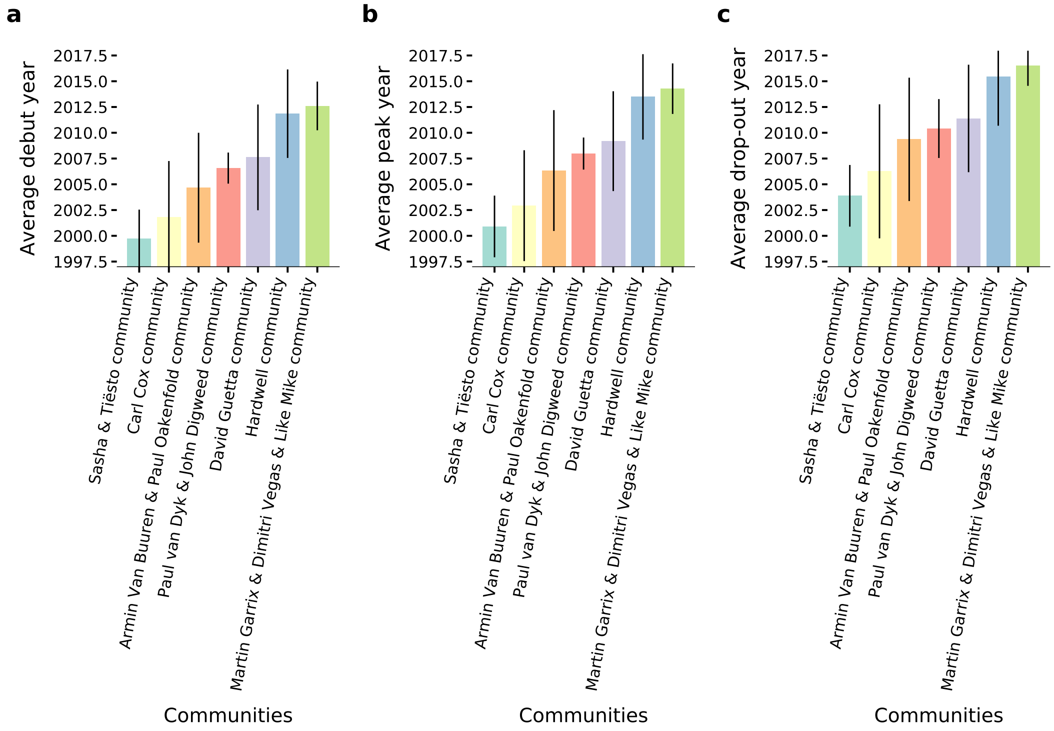

Figure S7. **Average time scales of DJ communities.** The average debut year, peak year (reaching the highest average rank), and drop-out years for the detected communities, different colors denoting the different communities.

S2.4 Typical timescales of DJ communities

We analyze the typical timescales of the different communities: the typical entry, drop-out, and peak years of the DJs belonging to all communities. As visualized in Figure S7, there is a clear temporal order between different communities. We also compare the distribution of entry years of DJs across communities (Figure S8), where we see that the community-leading figures have typically entered the ranking at the early stages of their communities' lifetime.

S2.5 Genre-similarities of DJ communities

In each DJ community a set of genres is present with varying degree. We encode this in the genre vector $\mathbf{g}_i$ for community i, were each element corresponds to a genre that was observed

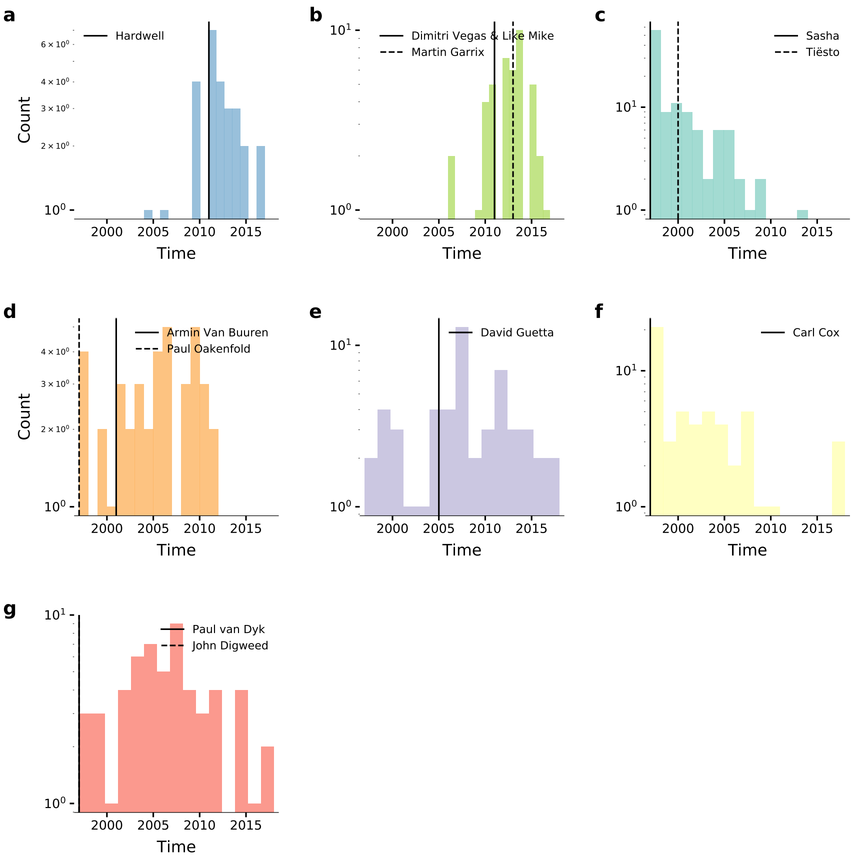

Figure S8. Entry rank distributions of DJ communities. Distributions of entry years of DJs in each community, with vertical lines for the entry years of each community's leading figures highlighting their early arrivals.

in the data set, and is equal to the number of times that genre occurred through the member artist profiles. For instance, if there are only two genres (e.g. techno and house music), and one community has 10 techno DJs, then their genre vector is $\mathbf{g} = (10,0)$. However, if a community has 5 techno and 5 house DJs, their genre vector is $\mathbf{g} = (5,5)$. After computing the observed genre vectors for the DJ communities, we measure their pairwise similarity based on the cosine similarity of their genre vectors, as introduced in the main text. These similarity results are shown in Table S3.

Paul van Dyk community	Armin Van Buuren & Paul Oakenfold community	0.842
Sasha & Tiësto community	Carl Cox community	0.821
David Guetta community	Martin Garrix & Dimitri Vegas & Like Mike community	0.764
Sasha & Tiësto community	David Guetta community	0.691
Hardwell community	Martin Garrix & Dimitri Vegas & Like Mike community	0.61
Sasha & Tiësto community	Armin Van Buuren & Paul Oakenfold community	0.566
Carl Cox community	David Guetta community	0.558
Carl Cox community	Armin Van Buuren & Paul Oakenfold community	0.489
David Guetta community	Armin Van Buuren & Paul Oakenfold community	0.444
David Guetta community	Paul van Dyk community	0.443
Hardwell community	David Guetta community	0.414
Paul van Dyk community	Martin Garrix & Dimitri Vegas & Like Mike community	0.413
Paul van Dyk community	Sasha & Tiësto community	0.391
Carl Cox community	Paul van Dyk community	0.354
Hardwell community	Paul van Dyk community	0.342
Martin Garrix & Dimitri Vegas & Like Mike community	Armin Van Buuren & Paul Oakenfold community	0.324
Hardwell community	Armin Van Buuren & Paul Oakenfold community	0.276
Sasha & Tiësto community	Martin Garrix & Dimitri Vegas & Like Mike community	0.224
Sasha & Tiësto community	Hardwell community	0.166
Hardwell community	Carl Cox community	0.166
Martin Garrix & Dimitri Vegas & Like Mike community	Carl Cox community	0.138

Table S3. Cosine similarity of the genre distribution of top DJ communities.

S2.6 Mentorship in electronic music

We extend the analysis presented in the main text to test several other hypotheses concerning mentee-mentor relationships and their effects on success.

We analyze the distribution of the number of times mentees collaborated with each mentor they had, showing a maximum at two collaborations (Figure S9a). This means that – surprisingly – mentees are most likely to work together with the same mentor twice. In other words, not ‘one-offs’ but ‘two-offs’ seem to be the most typical pattern of collaboration in electronic music.

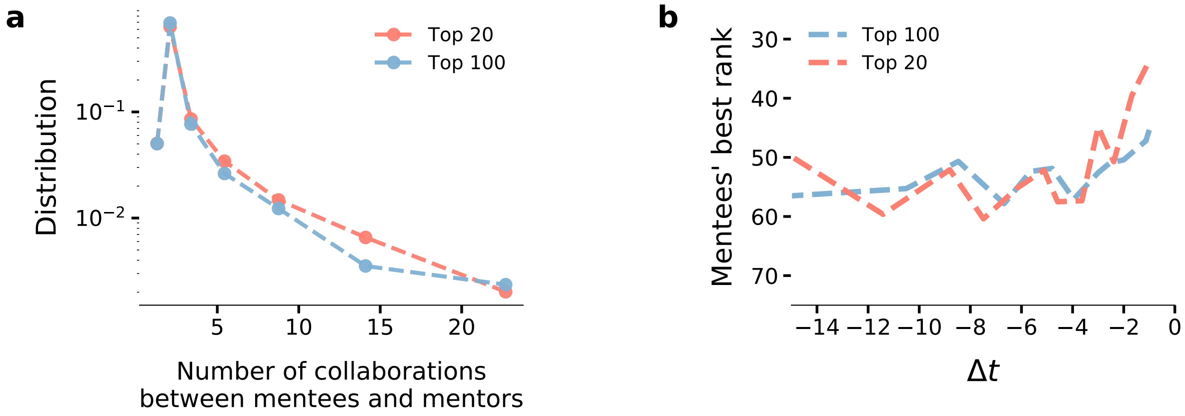

Figure S9. Mentee-mentor relationships. (**a**) Binned distribution of the number of times mentored DJs worked with the various mentors they had. (**b**) Average mentees' best rank as a function of entry-time difference Δt (experience gap) between every possible mentor and mentee pair.

We also note that three- and four-time collaborations between mentors and mentees happen more often than single collaborations on average.

We also test whether the experience gap between mentors and mentees has an effect on the best rank of mentees. We define this experience gap as the entry-time difference between each pair of mentor and mentee who worked together:

$$\Delta t = t_{\text{mentor}} - t_{\text{mentee}}. \tag{2}$$

Our results are illustrated in Figure S9b, which shows that the best rank of mentees is relatively insensitive to the entry-year difference with their mentors (at least when $|\Delta t|$ is large). Indeed, the Spearman correlation between the entry-year difference of mentors and mentees and the mentees' best rank for $\Delta t < -4$ is ~ 0.030 for top 20 mentors and ~ 0.027 when we consider every DJ. However, for $\Delta t > -5$ we see a slightly stronger correlation of ~ 0.275 for top 20 DJs and ~ 0.121 for all the DJs included. These results show that only smaller entry-time (i.e. experience)

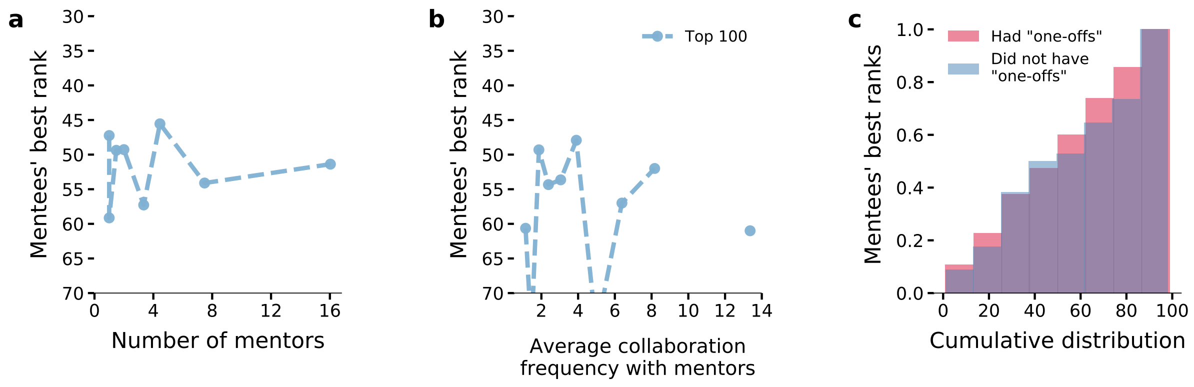

Figure S10. Collaboration characteristics and mentee success. Mentees' best ranks as a function of (**a**) the number of mentors they have had during their career, and (**b**) the average frequency of collaboration with their mentors. (**c**) Comparison of the cumulative best-rank distribution of mentees, based on whether they did or did not have one-off collaborations during their career.

differences boost the success of mentees, which we suspect is more related to the effect of joining trending communities than an effect of the mentors themselves (this would be consistent with our previous findings as well).

We compare the mentees' best rank to the number of mentors they have had during their career (Figure S10a), finding marginal (< 0.05) correlations between the two quantities. We also investigate the relationship between the mentees' best ranks and the average number of times they have collaborated with their mentors (Figure S10b). Results show that "one-off" collaborations bring low success as their associated best ranks are just around 60. Yet, there seems to be a sweet spot – a peak in Figure S10b around rank 40 showing that collaborating with mentors three or four times on average can help mentees make it up to rank 40. Finally, we explicitly characterize "one-off" collaborations and find that only 17% of the mentored DJs have had such a collaboration. There is no significant difference between the best rank distributions of DJs with or without "one-off" collaborations (Figure S10c). We compare the distributions

by using a double-sided Kolmogorov-Smirnov test and find the estimated KS distance to be $d \approx 0.155$ with a significant value of $p \approx 0.43$.

S2.7 Online resources and data

All data used in this study is openly accessible from the following online resources: DJ Magazine[3] and its top 100 ranking,[4] the Amsterdam Music Festival,[5] Discogs,[6] crowdsourced collections of DJ Magazine top 100 rankings,[7,8] LastFM,[9] and Billboard Magazine.[10]

References

1. Coscia, Michele and Neffke, Frank MH Network backboning with noisy data. *Proc Int Conf Data* **14**, 425–436 (2017).
2. Blondel, Vincent D and Guillaume, Jean-Loup and Lambiotte, Renaud and Lefebvre, Etienne Fast unfolding of communities in large networks. *J Stat Mech-Theory E* **14**, P10008 (2008).
3. Djmag, Djmag.com (2019).
4. Djmag Top 100, djmag.com/top-100-djs (2019).
5. Amsterdam music festival, amf-festival.com (2019).
6. Discogs, discogs.com (2019).
7. Crowdsourced collection of djmag top100 rankings, vk.com/topic-4286148_28357370.
8. Crowdsourced collection of djmag top100 rankings, edm.fandom.com/wiki/DJ_Mag_Top_100_DJs.
9. Lastfm, last.fm (2019).
10. Billboard, billboard.com (2019).
